# Efficacy and Safety of Statin Treatment in Children with Familial Hypercholesterolemia: Outcomes of 20 Years of Experience

**DOI:** 10.3390/jcm12237197

**Published:** 2023-11-21

**Authors:** Radosław Motkowski, Paweł Abramowicz, Jolanta Kubalska, Bożena Mikołuć, Jerzy Konstantynowicz

**Affiliations:** 1 Department of Pediatrics, Rheumatology, Immunology and Metabolic Bone Diseases, Medical University of Bialystok, 15-274 Bialystok, Poland; pawel.abramowicz@umb.edu.pl (P.A.); bozena.mikoluc@umb.edu.pl (B.M.); jurekonstant@o2.pl (J.K.); 2 Department of Genetics, Institute of Psychiatry and Neurology, 02-957 Warsaw, Poland

**Keywords:** atherosclerosis, statins, children, familial hypercholesterolemia, safety

## Abstract

Background: The objective of this retrospective cohort study was to present the experience of 20-year-long comprehensive care of pediatric patients with familial hypercholesterolemia (FH) in a single academic center. Methods and Results: The study included 84 children aged 1–18 years with FH. For the whole study group, 535 medical visits were recorded. The mean follow-up period was 33.6 months. Molecular testing performed in 55 children (65%) provided genetic confirmation of the diagnosis in 36 children (43%). Twenty-seven children (32%) were treated pharmacologically with statins. Follow-up during the treatment averaged 29 months. Treatment with statins was associated with a mean reduction in total cholesterol and LDL-cholesterol levels of 24 and 33% from the baseline. Symptoms of statin intolerance occurred incidentally and did not require amendment in the treatment protocol. Significantly higher values of body weight, height, and BMI were found only among girls older than 10 years who were treated with statins. Conclusions: These data confirm a high efficacy and a good safety profile of statin treatment in children with FH, demonstrating no harm to physical development. However, there is a need for further cause-and-effect research regarding associations between long-term treatment with low-cholesterol, low-fat diets, statin therapy, and excessive weight gain.

## 1. Introduction

Familial hypercholesterolemia (FH) is a congenital metabolic disorder (OMIM #143890, #144010, #107703, #607786), which is connected to increased serum low-density lipoprotein cholesterol (LDL-C), leading to premature atherosclerosis and diseases of the cardiovascular system [1]. The average age of the first hospitalization due to cardiovascular disease in this group of patients is 45 years, i.e., approximately 20 years earlier than in the general population [2]. The incidence of familial hypercholesterolemia in Europe has been estimated at 1:217 according to recent studies [3]. In contrast, the mortality due to circulatory system diseases in these patients is 100 times higher compared with the general population [4,5]. Lipid-lowering treatments initiated early significantly reduce the risk later in life [6,7].

According to current clinical practice, the most common hypolipemic therapeutic management used among FH children is statins [8]. The safety and efficacy of statin treatment in children were evaluated a couple of years ago in several meta-analyses [9], including those incorporated in the Cochrane Reviews Evidence [10]. Unfortunately, these reports concerned only studies with a maximum follow-up duration of 2 years, thereby suggesting a necessity for longer and well-designed observations in this specific group. In an analysis for the Cochrane Library, the importance of the careful monitoring of lipid parameters, the incidence of adverse effects, other risk factors of atherosclerosis, and child growth and development during statin treatment [10].

Existing evidence on the efficacy of statin therapy renders it ethically impossible to design a long-term randomized trial. Further, it is all the more essential to monitor and follow-up on children already undergoing hypolipemic treatment concerning treatment tolerance, several aspects of statin safety, and the children’s development [11].

The recent literature encompassing longer follow-ups have usually reported aggregated data originating from many centers, including a 5-year follow-up of the efficacy and safety of the treatment in a UK cohort of 232 children [12], Norwegian data covering 302 children [13] and also combined data from Canada and the United States covering 289 pediatric patients [14].

Specific evidence for the effectiveness of statin treatment was provided by a 20-year follow-up from The Netherlands [15]. This report did not compare children treated vs. not treated with statins, but children with FH treated with statins and their healthy siblings and affected parents. The researchers showed that the risk of cardiovascular disease before the age of 39 for untreated parents was 26% (7% died during that time), whereas for their children treated from the age of 13, it was 1%. Moreover, the early initiation of statins in children with FH alleviated the progression of carotid intima-media thickness [15].

A parameter called Cumulative LDL-C burden is used to determine the risk of developing ischemic heart disease. The level of 160, associated with the development of ischemic heart disease, is reached by a person without FH at the age of 55. In contrast, a patient with heterozygous FH reaches it at the age of 35 years. If lipid-lowering therapy in an individual with FH is initiated at 18 years, the cumulative LDL cholesterol burden of 160 will be achieved at the age of 48. If the treatment with statins is initiated at the 10th year of life, a similar cumulative LDL cholesterol burden will be reached around the age of 53 years. [7].

This retrospective cohort study aimed to present the experience of 20 years of care in pediatric patients with familial hypercholesterolemia in a single specialist academic center. The efficacy and safety of the hypolipemic treatment with statins were reviewed in detail, especially in the context of growth and physical development measured with basic anthropometric parameters.

## 2. Materials and Methods

### 2.1. Study Participants

The study included 84 children (42 girls) aged 1 to 18 years who were consulted at least twice between 2001 and 2021 at the Metabolic Outpatient Clinic of the University Children’s Hospital in Bialystok, Northeastern Poland, for familial hypercholesterolemia diagnosed using the Simon Broome Criteria [16]. For the whole study group, 535 medical visits were recorded, with an average of 6 for each patient (range: 2–27). The mean follow-up time was 33.63 months (SD 37.3), the shortest 2 months, and the longest 173 months (14.5 years). All children in this study had a family history of hypercholesterolemia and/or premature cardiovascular disease. Molecular testing was performed in 55 children (65%). The tests revealed the presence of a known gene mutation underlying the diagnosis of FH in 36 children (43%). In 19 children, molecular testing failed to identify a known mutation in the LDL receptor or apolipoprotein B genes.

A low-cholesterol diet, with restrictions on saturated and trans fats, was recommended to all studied children, and the rules were consistently reminded to the children and caregivers at each follow-up visit. Cholestyramine treatment was attempted in five children between 2005 and 2010 and was discontinued due to intolerance of the form and taste of the preparation. Twenty-seven children (32%) were treated pharmacologically with statins. Treatment was usually implemented at the 4th visit, after an average follow-up of 34 months (SD 29.26; min. 3 months, max. 100 months), with a mean age of 13 years (SD 2.47; range: 8.5–17 years). The first follow-up visit was held after two months (1–3 months) and the follow-up time during treatment averaged 29 months (SD 24.27; the most extended observation was 75 months). A total of 18 participants (66%) were treated with simvastatin at the time of the last visit (twelve subjects with a dose of 10 mg, six subjects with 20 mg), and 9 participants were receiving rosuvastatin (6 with a dose of 5 mg, 2 with 10 mg, and 1 with 20 mg).

The remaining 57 children (68%) did not receive pharmacological treatment due to age limitations associated with the registration and product characteristics for hypolipidemic drugs (8 years for rosuvastatin and 10 years for simvastatin) or because of a lack of parental consent. Detailed characteristics of children treated with the diet and statins and those treated with the diet alone are shown in Table 1.

### 2.2. Methods

The study was based on a retrospective review of medical records. Anthropometric data and clinical features (body weight, body height, heart rate, and blood pressure values), as well as available biochemical test results (lipid profile, glucose level, creatinine kinase, and aminotransferase activity), were included in the study. Data on body weight, height, and a standard formula for BMI were also cross-referenced with reference values for age- and sex-matched normative values (growth charts, Z-score).

We also analyzed recorded information on chronic complaints, concomitant diseases, co-morbidities, and, in the group of children treated with statins, treatment tolerance (i.e., occurrence of adverse reactions and side effects). The protocol was approved by the Bioethics Committee at the Medical University of Bialystok. The study was supported by the Medical University of Bialystok No. SUB/1/DN/19/001/1126.

Blood for testing was collected in the morning after 12 h of fasting. Glucose was determined using the enzymatic hexokinase/glucose 6-phosphate dehydrogenase method (COBAS 6000 C501, Roche, Mannheim, Germany). Lipids (i.e., total cholesterol, LDL- and HDL-cholesterol, and triglycerides), alanine and aspartate aminotransferase activities, and creatinine kinase were measured with the colorimetric enzymatic method (COBAS 6000 C501, Roche, Mannheim, Germany).

Molecular tests were performed using the PCR RFLP method and MLPA sequencing, and in some patients, the next-generation sequencing method (NGS) on the MiSeq platform (Illumina Inc., San Diego, CA, USA) using the ADH MASTR (Multiplicom, Niel, Belgium) kit. The tests were performed at the Institute of Psychiatry and Neurology in Warsaw and the University Clinical Center of the Medical University of Gdańsk.

Statistical analysis was performed using the arithmetic mean and standard deviation (SD) for quantitative variables. The Chi^2^ test assessed the significance of differences in qualitative variables. The examined continuous variable distribution was evaluated with the Kolmogorov–Smirnov test. For the analysis of the variables distributed normally, the Student’s *t*-test was used, and for the variables inconsistent with the normal distribution, the Mann–Whitney U test was used. Stepwise multiple regression analysis was conducted to determine relationships between the selected variables included in the models.

The anthropometric parameters converted to Z-scores were calculated separately for subgroups stratified by age and sex (with a 1-year range precision) based on population data published elsewhere [17]. The following standard formula was used to determine the Z-score: Z-score = (parameter—SD for corresponding age and sex)/median for corresponding age and sex.

In the calculations, the level of *p* < 0.05 was accepted as statistically significant, authorizing the rejection of individual null hypotheses. The data were processed using the Polish version of Statistica 13.0 statistical software for Windows PCs. Raw data used in the publication are available in Supplementary Materials.

## 3. Results

### 3.1. Lipid Profile

**Figure 1:**
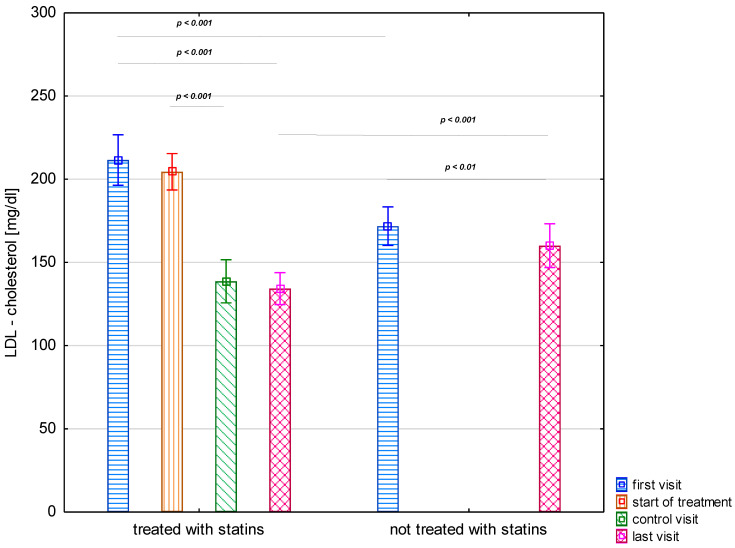
LDL lipoprotein cholesterol levels in the study group stratified by statin treatment.

In the study group, the mean total cholesterol at the first visit was 258 mg/dL (SD 42.23), and LDL-C was 185 mg/dL (SD 43.93). Total cholesterol and LDL-C levels found at the beginning of the follow-up in the FH children who later received statin treatment were higher compared with peers who did not receive such treatment later (Table 2, Figure 1). After treatment with the dietary regimen alone, a similar decrease in total cholesterol was observed in both groups, with a mean reduction of 5.27% in the group subsequently treated with statins and 4.68% in the untreated group, and a decrease in LDL-C by 4.45% and 13.7%, respectively. Only in the group of children who had never been treated with statins were these differences significant (*p* < 0.001).

Treatment with statins was associated with a mean reduction of 24 and 33% from the baseline in total cholesterol and LDL-C levels after just two months (control visit) (Table 2, Figure 1). Subsequent months of treatment resulted in further reductions in total cholesterol and LDL-C, and during the entire follow-up, the values were lower by 27% and 36% than the baseline, respectively. 

Based on records from the most recent available visits, total cholesterol and LDL-C concentrations in the children treated concurrently with the diet and statins for a mean follow-up of 29 ± 24.26 months were significantly lower than for children treated with diet alone after 29 ± 28.76 months of follow-up (Table 2, Figure 1). At the last recorded visit, 14 (52%) of the statin-treated children still had LDL-C levels above 130 mg/dL, but only 2 (7%) had levels above 160 mg/dL. In the group of children not treated with statins, LDL-C levels above 130 mg/dL were observed in 38 (66%) children, and those above 160 mg/dL were found in almost 1 in 3 children (20 of 57–35%). 

There were no differences in HDL lipoprotein cholesterol and triglyceride levels, except for a slight decrease in HDL lipoprotein cholesterol during statin treatment (Table 1). The decline in HDL lipoprotein cholesterol levels during the entire follow-up averaged 7.7% in this group and averaged 10% in the diet-only treatment group. Triglyceride levels in the diet-only children decreased by 4%, whereas, in the statin-treated group, the TG levels eventually reduced by an average of 3%.

### 3.2. Comorbidities and Tolerability of Statin Treatment

During each of the 535 analyzed medical consultations, caregivers of children with hypercholesterolemia were asked questions about comorbidities and the tolerance of treatment. Two patients dropped out of further treatment and lipid care after attempting oral cholestyramine. Coexisting celiac disease and hypothyroidism were found in two patients.

The 27 children treated with statins were consulted 315 times. Two boys were diagnosed with scoliosis during puberty, and one boy developed avascular necrosis of the hip. One patient was diagnosed with an ovarian cyst and obesity; another female patient showed overweight, hypertension, and menstrual disorders. One boy was diagnosed with congenital macular dystrophy, another one had psoriasis, and one girl was diagnosed with epilepsy during follow-up.

One patient experienced a transient increase in total cholesterol after being introduced to retinoids for severe acne lesions. Another patient was switched to rosuvastatin due to intolerance to simvastatin (headache, weakness, fatigue). In one boy, due to lack of parental consent, the treatment was discontinued for 6 months because of exacerbated psoriatic lesions that coincided with the introduction of simvastatin treatment. No progression of skin lesions was then observed after restarting treatment.

Nine children had fasting blood glucose values > 100 mg/dL in single measurements, but only one boy had a fasting blood glucose greater than 120 mg/dL. Three children had single glycemic values < 60 mg/dL. No child was diagnosed with diabetes.

Only one boy with hepatic steatosis of unknown etiology, diagnosed prior to treatment, had elevated aminotransferase activity. After achieving target LDL-cholesterol values with normal aminotransferase activities, the patient discontinued treatment and care at the Metabolic Clinic.

Four patients had increased creatinine kinase activity, all of which was due to the test being performed the day after excessive exercise. The values exceeded five times the upper limit in only two of them. The parents of one of these patients, whose molecular testing did not reveal the presence of a pathogenic mutation, waived further drug treatment. Another patient with a strong family history and a confirmed mutation in the LDL receptor gene returned to treatment with the previously used dose of rosuvastatin. With the rule of one day’s rest from sports, before the test was performed, he was never again found to have increased creatinine kinase activity afterward.

Nine children showed heart rates higher than 90/min two or more times, and one boy showed bradycardia below 60/min. Blood pressure values were recorded during 176 visits and averaged 116.7 mmHg and 71.3 mmHg systolic and diastolic, respectively (75–95 centile according to standard reference). Two or more abnormal systolic blood pressure measurements (>120 mmHg) were found in 10 children. Five children also demonstrated elevated diastolic blood pressure values (>80 mmHg). Two of them were not treated with statins due to being too young at the time of the study. In all of these children, further diagnostics confirmed the diagnosis of so-called white coat hypertension.

### 3.3. Anthropometric Measurements

**Figure 2:**
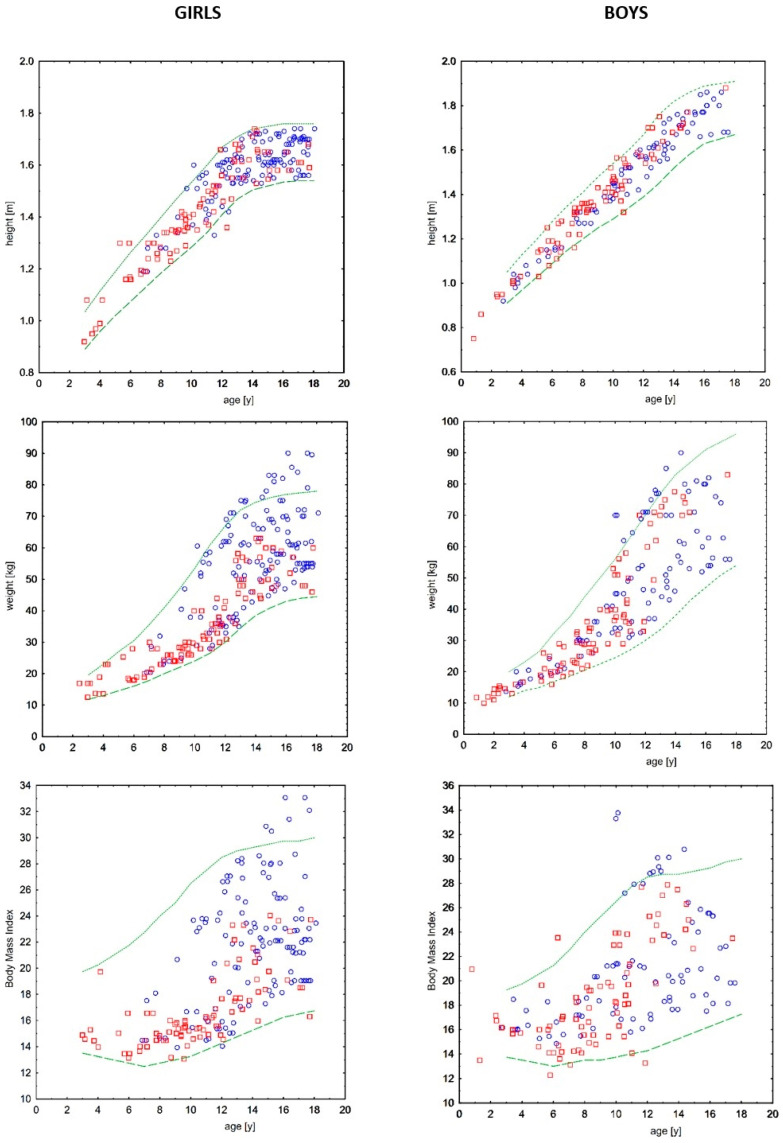
Anthropometric parameters in studied children with familial hypercholesterolemia. Circles—treated finally with statins; squares—never treated with statins; dashed line—3rd centile; dotted line—97th centile according to the standard reference from OLAF study [17].

The distribution of body weight, height, and BMI of the male and female participants in relation to the standard reference is shown in Figure 2 (3rd and 97th centile according to OLAF) [17]. The body height remained within normal limits. There were no deficiencies in body weight throughout the follow-up period. The summary data presented in the graph revealed a higher prevalence of weight above the age standard (97th centile) in older children, more prevalent among girls, and more frequent among children treated with statins. The prevalence of obesity, defined as a BMI above the 97th percentile, was found less frequently but still more often among older, mainly female children and those treated with statins.

The Z-score values for body weight in participants treated with statins were statistically significantly higher than in the children treated with diet alone, averaging 1.13 (SD 1.49) vs. 0.49 (SD 1.15). A similar relationship was found by assessing the Z-score values for BMI, with an average of 0.98 (SD 1.38) vs. 0.25 (SD 1.15). Differences in the body height Z-score were not significant.

A multiple regression analysis was performed to determine the significance of the differences regarding anthropometry (height, weight, and BMI) as dependent variables, and age, sex, total cholesterol, and statin treatment as independent variables. There was an association of body height only with age (R 0.93) and sex (R 0.1), as well as body weight with age (R 0.86), sex (R 0.15), TC levels (R −0.08), and statin treatment (R −0.12). The BMI values depended less on age (R 0.55), sex (R 0.19), and total cholesterol (R −0.07) but more distinctly on the statin treatment (R −0.22) in this model.

Based on the above results, the differences in the anthropometric parameters between children treated with statins and those treated with diet alone were stratified by age and sex subgroups (children younger or older than 10 years). The age threshold we adopted was arbitrarily determined based on two rationales—the only existing comparable report [12], which also adopted the age of 10 years as a criterion for stratification, and the registration restrictions of simvastatin (the most commonly used statin), which has been approved for pediatric treatment in individuals aged over 10 years.

There were no significant differences among boys except for a slightly greater height in older boys treated with statins. There were no significant differences between the younger girls either. In contrast, significantly higher values of body weight, height, and BMI were found among girls older than 10 years who were treated with statins compared to those who were treated with the diet alone. The analysis of anthropometric measurements related to sex and age (Z-scores) did not confirm differences in body height but again showed higher Z-scores for body weight and BMI in the group of statin-treated girls over 10 years of age (Table 3).

## 4. Discussion

This study summarizing a 20-year-long follow-up of a pediatric population with FH showed that statin therapy, when introduced reasonably early, is safe, provides benefits, and does not cause health hazards during growth. Importantly, there is still a limited number of published data on statin use in children and adolescents and related associations.

The mean age of children who had been treated later with statins was higher at the time of their first visit to our center than the mean age of children in whom treatment was not initiated. A similar relationship and almost identical values were observed in the Norwegian cohort [13]. There is no such direct data in the UK report, and the mean duration of treatment in the North American study was 2.7 years [14]. The time to introduce statins into treatment was later than recommended (10 years of age) in all populations: 11 years in the UK, 12.5 years in Norway and North America, and 13 years in our center. As reported elsewhere, this is due to a delayed diagnosis associated with cascade screening in these countries and a lack of population-based screening [18].

In a study conducted in Norway, children treated with statins demonstrated a 38% reduction in LDL-C between the first and last visits, whereas children not treated with statins had 4% [13]. In a similar study from the UK, this difference was 35% [12]. A recent report encompassing data from eight European countries described reductions in LDL-C of 28% to 57%. However, when data from Greece, where population-based screening was conducted between the 1st and 3rd years of age, were excluded from the analysis, the mean reduction in LDL-C during statin treatment was much greater, i.e., 33.5% [18]. Another study from Poland showed a 34.4% decrease in LDL-C following one-year treatment with rosuvastatin, although at different doses [19].

In our study, we observed a similar efficacy in children treated with statins. We reported a 33% reduction in LDL-C levels after just two months and 36% during the entire follow-up. We found a concomitant more significant (13.7%) decrease in LDL lipoprotein cholesterol levels in the group on long-term diet and lifestyle modification than in the Norwegian (4.5%) or the UK (5.4%) studies. This may be similar to an option of dietary correction of lipid disorders in children, as described in the literature, ranging from 10 to 15% [20,21]. We hypothesized that such an outcome may have resulted from regular consultations on low-fat, hypolipemic diet rules and also the involvement of both parents and children. Other studies have no data on the frequency of dietary counseling.

In the above-mentioned Norwegian study, a higher proportion of statin-treated children achieved target LDL-C levels than in our study (71% vs. 48%). In the study by Ramaswami et al., the percentage of children whose LDL-C levels fell below 130 mg/dL was 54% after excluding data from Greece, where up to 99% of children achieved the therapeutic goal [18]. In the study from North America, 51% of studied children failed to reach this strategic goal [14]. Notably, our results (like those in other studies on pediatric populations) were much better compared with some published studies among adults, where only 2.7% reached the target concentration [22]. In an interesting Norwegian paper, 22% of young adults reached the treatment goal, and 30% of children and young adults experienced treatment adherence problems [23]. We found more significant differences in children who did not receive pharmacological treatment. In the Norwegian study, 41% of such children did not reach the therapeutic goal, while in our study, 66% did. Unfortunately, significantly higher LDL-C levels than the target (>160 mg/dL) were present in up to 35% of our participants. No such data are available from other studies.

To date, there are no evidence-based recommendations addressing the degree of LDL-C reduction in children with FH that would effectively prevent the development of atherosclerosis and cardiovascular disease. However, the 32% reduction in LDL-C described in a Dutch longitudinal observational study is comparable to the effect obtained in most studies described above, so it reduced the risk of early cardiovascular disease from 26 to 1% [15]. This reduction in LDL-C was possible in our studied children after statins were used in monotherapy, at the lowest available doses, and in a short time—following approximately two months of intervention.

Regarding our patients with FH, we included data on comorbidities found in a number of these subjects during a longstanding follow-up, up to 15 years in some cases. Associations between coexisting conditions and events with the treatment cannot be ruled out, nor can they be confirmed. Available data do not report such diseases occurring in association with statin treatment in children or even in extensively studied adult populations. The most common reports of adverse effects attributable to statin treatment involve abnormal muscle and liver enzymes and the occurrence of diabetes. A Cochrane Library meta-analysis of nine studies involving 1177 children treated with statins for 4 to 24 months found incident adverse events only. The incidence was not different between children treated with statins and those receiving placebo [10].

In the Norwegian report, 5% of the children had adverse effects but were not associated with biochemical abnormalities. We found increased, though unrelated to treatment, aminotransferase activity in one patient, whereas none of the Norwegian studies showed such an abnormality. In both studies, an increase in creatinine kinase activity associated with increased exercise was observed in several children. Of importance, no one experienced symptoms of myopathy or rhabdomyolysis [13]. In the UK study, none of the participants showed increased creatinine kinase or aminotransferase activities [12]. In a report from Canada and the US, 5% (*n* = 15) of the children had increased creatinine kinase activity, but none had clinical symptoms, thus, no treatment modification was required. Increased aminotransferase activity was observed in 4%, which was normalized after the discontinuation of therapy and did not increase when treatment was restarted. Twenty patients (7%) experienced muscular pain, fatigue, rash, and abdominal pain but did not require a change in treatment [14]. In the Dutch cohort, there were no statistically significant differences in liver enzymes and creatinine kinase activity between statin-treated children with FH and their healthy siblings [15].

In our study, as reported elsewhere, symptoms of statin intolerance occurred incidentally and usually did not require amendment in the treatment protocol. In the adult population, such symptoms are equally rare (5 to 10%) but more often require treatment modification of the dosage, change of the active agent, or introduction of polytherapy to the treatment [24]. A recent meta-analysis of 62 studies involving more than 120,000 adults found that treatment with statins was associated with a small but significant risk of muscle complaints or liver dysfunction. These manifestations were mostly transient, depended on the type and dose of statin used, and also occurred in the placebo group [25]. Muscle damage occurs in less than 0.1% of adults, and the risk of liver damage occurs in 0.001%. When assessing the potential risk of adverse effects, the absolute benefits of statin therapy in preventing cardiovascular disease should be kept in mind, e.g., a 10% reduction in mortality with each 39 mg% reduction in LDL-cholesterol [21].

We also evaluated other atherosclerosis risk factors to determine target LDL-C levels while monitoring treatment for lipid disorders. None of the children in our study were diagnosed with hypertension or diabetes. No patient admitted to smoking cigarettes either. Nevertheless, more attention was needed to monitor the prevalence of overweight and obesity.

To our knowledge, only aggregate data on mean weight and height, or BMI, of children with hypercholesterolemia treated with statins are available in the current literature. None of our studied children were malnourished or short-statured during treatment. Obesity, defined as body weight or BMI > 97th centile for sex and age, was also less frequent in the entire group than in the general population. However, a detailed analysis of available data from 535 follow-up visits and their reference to reference charts showed a higher prevalence of obesity among children treated with statins (Figure 2). A multiple regression analysis confirmed that body weight and BMI were related to statin treatment, and a subgroup analysis showed that obesity was significantly more common in statin-treated girls older than ten years.

In the British report, children did not differ in weight or height at diagnosis. The anthropometric parameters assessed at the last follow-up visit were higher in those who started treatment before the age of 10 years compared with those not treated. No such differences were found in the group of children who began treatment after the age of 10 years [12]. In the North American study, the mean body weight centile before treatment implementation was 84 (ranging from 51 to 96), and it was 86 (53–97) at the last follow-up visit, but this difference was not significant. The mean BMI centile before treatment initiation was 89 (62–98), and it was 87 (54–97) after treatment [14]. The Dutch cohort showed a marked change in BMI from 19.6 to 25.4 after several years of treatment, but these results were unrelated to centile charts or Z-scores [15]. Medeiros et al. found that 14.1% of patients with FH had a BMI above the 95th percentile compared to 33.8% of patients with hypercholesterolemia of other etiologies [26].

The interpretation of the relationship found in our study is hampered by the lack of comparable reports that would have included multiple assessments of anthropometric parameters over a more extended follow-up period. Based on clinical experience in pediatric patients with FH, an explanation is that patients and their families often discontinue a low-cholesterol diet after the introduction of pharmacotherapy has been initiated. Every patient at our center was reminded during follow-up visits of the low-cholesterol diet, with a restriction of saturated fats, and was advised to maintain appropriate physical activity. Unfortunately, in many cases, numerous errors were reported by parents. What is challenging and questionable is the lack of a significant increase in body weight among boys who were more likely to make dietary mistakes than adolescent girls. Perhaps this may be related to boys’ greater physical activity during puberty. However, it is noteworthy that obesity is an independent risk factor for atherosclerosis and the development of cardiovascular disease. Studies among adults have not found a higher incidence of obesity in people treated with statins, but this may be related to the age at which treatment began [27].

Some recommendations have indicated only the supportive nature of dietary treatment in these patients [4,5]. A lack of evidence for the advisability of low-fat and low-cholesterol diets in patients with FH has also been raised, however, at the same time, low-carbohydrate diets’ potential benetifs have been pointed out [28]. The explanation of the phenomenon described above requires further well-designed prospective studies. However, regardless of the reasons for the observed tendency to increase body weight during drug therapy, dietary monitoring should remain an essential part of care in children with FH [1]. Consistent dietary counseling can be critical in preventing overweight and obesity [27].

A limitation of our study is its observational and retrospective nature. In addition, the number of patients enrolled was relatively small compared to the expected prevalence since FH remains underdiagnosed and undertreated in children. Notably, the average age of diagnosis for FH is 44 years, and only 2.1% of those affected are diagnosed in their teens [22]. Another limitation is that only a limited proportion of all participants had a molecular diagnosis. However, the initiation of treatment was not restrictively determined by the result of a genetic test. Furthermore, the availability of the method was very limited at those times, i.e., during the first years of our follow-up. All children included in the study were diagnosed with FH using recognized clinical criteria.

We had no control over the disruptions that were associated with some children remaining in care for extended periods and some children dropping out of care early. Further research is also needed to assess the reasons why some parents opt for drug treatment for their children, whereas others do not. On the other hand, the strength of our study is the long follow-up duration and the large number of follow-up visits analyzed. Furthermore, the unified management of a child and the parents for a child with hypercholesterolemia within a single center, as well as the amount of routinely collected data, including anthropometric data, are noteworthy.

Atherosclerotic cardiovascular disease occurs worldwide and is regarded as the most common cause of death in adults. Many of these conditions could be prevented by diagnosing FH early enough. Unfortunately, although the disease is the most common monogenic condition, it is undoubtedly underestimated and diagnosed only in a small proportion of affected individuals and usually only after the onset of clinically apparent atherosclerosis [29]. The hypolipemic treatment used in secondary prevention would always be more complicated than that in primary prevention. An effective treatment initiated in childhood can significantly extend the healthy life span of patients with FH.

The results of our study presenting 20 years of experience from only one center confirm a high efficacy, good safety profile, and overall benefits of statin treatment in children with familial hypercholesterolemia. These data support the view of the necessity of the early implementation of statin therapy for pediatric patients with FH. There is a need for further cause-and-effect research regarding associations between long-term treatment with low-cholesterol and low-fat diets and excessive weight gain during pharmacotherapy.

## Figures and Tables

**Table 1:** Characteristics of hypercholesterolemic groups. Data are presented as means and standard deviations, where applicable.

	Treated with Statins (*n* = 27)	Without Statins (*n* = 57)	*p*
Age at baseline [y] during the first visit	10.03 (3.57)	8.09 (3.60)	0.009
Sex	16 girls, 11 boys	26 girls, 31 boys	0.243
Family history of hypercholesterolemia:			
Siblings	17 (63%)	25 (44%)	0.102
Parents	27 (100%)	52 (91%)	0.113
Grandparents	19 (70%)	47 (82%)	0.491
Mutation status:			0.001
- LDL-R	15 (56%)	12 (21%)	
- APO-B	7 (26%)	2 (4%)	
- neither LDL nor APO-B	2 (7%)	17 (30%)	
- not performed or under testing	3 (11%)	26 (46%)	
Time of observation [months]	64.44 (42.61)	29.75 (28.76)	0.001
Number of visits	11.67 (5.21)	3.88 (2.77)	0.001
Age [y] during the last visit	15.63 (2.36)	10.55 (3.68)	0.001

**Table 2:** Lipid profile in study groups during follow-up. Data are presented as means and standard deviations in parentheses. The letters in the superscript indicate statistically significant differences from *p* < 0.001. The symbols in the superscript indicate significant differences from *p* < 0.01.

	Treated with Statins (*n* = 27)				Without Statins (*n* = 57)	
	First Visit	Start of Treatment	Control Visit	Last Visit	First Visit	Last Visit
Total cholesterol [mg/dL]	284.00 ^a,e^ (35.97)	279.23 ^k^ (30.36)	213.11 ^k^ (36.65)	204.26 ^c,e^ (26.30)	245.37 ^a,#^ (39.43)	232,96 ^c,#^ (46.56)
HDL cholesterol [mg/dL]	58.29 (23.88)	59.88 (18.64)	60.56 ^$^ (14.42)	55.54 ^$^ (13.57)	56.93 (13.37)	55.37 (11.49)
LDL cholesterol [mg/dL]	211.53 ^b,f^ (37.56)	204.50 ^m^ (27.14)	138.63 ^m^ (32.78)	134.27 ^d,f^ (23.78)	171.80 ^b,%^ (41.03)	160.06 ^d,%^ (46.72)
Triglycerides [mg/dL]	86.42 (52.59)	81.20 (35.57)	73.78 (29.80)	71.84 (26.26)	94.66 (63.88)	87.15 (38.88)

**Table 3:** Anthropometric parameters in study groups divided according to sex and age. Data is presented as means and standard deviations in brackets. The quantity *n* denotes the number of available measurements.

			Treated with Statins *n* = 232	Without Statins *n* = 195	*p*
Boys					
<10 years	Weight *n* = 86	[kg]	27.46 (11.41)	24.39 (9.45)	0.19
		Z-score	1.05 (1.31)	0.66 (1.14)	0.21
	Height *n* = 75	[m]	1.21 (0.15)	1.22 (0.17)	0.62
		Z-score	0.56 (0.81)	0.89 (1.11)	0.11
	BMI *n* = 75	[kg/m^2^]	17.96 (3.48)	16.83 (2.69)	0.07
		Z-score	0.92 (1.27)	0.41 (1.29)	0.06
≥10 years	Weight *n* = 98	[kg]	59.40 (15.43)	54.28 (17.05)	0.14
		Z-score	1.14 (1.57)	1.41 (1.24)	0.24
	Height *n* = 88	[m]	1.63 (0.12)	1.57 (0.13)	0.04
		Z-score	0.75 (0.84)	1.06 (0.96)	0.12
	BMI *n* = 88	[kg/m^2^]	22.3 (4.44)	22.87 (4.16)	0.81
		Z-score	1.15 (1.56)	1.29 (1.22)	0.29
Girls					
<10 years	Weight *n* = 60	[kg]	29.38 (8.89)	23.59 (5.49)	0.08
		Z-score	0.62 (1.17)	0.26 (1.09)	0.41
	Height *n* = 48	[m]	1.31 (0.10)	1.25 (0.13)	0.45
		Z-score	0.69 (1.02)	0.64 (1.27)	0.92
	BMI *n* = 48	[kg/m^2^]	16.12 (2.27)	14.93 (1.23)	0.29
		Z-score	0.23 (1.05)	−0.39 (0.70)	0.16
≥10 years	Weight *n* = 183	[kg]	58.26 (14.09)	44.83 (10.47)	0.01
		Z-score	1.19 (1.53)	0.02 (0.81)	0.01
	Height *n* = 161	[m]	1.61 (0.09)	1.56 (0.10)	0.01
		Z-score	0.34 (1.1)	0.19 (1.12)	0.39
	BMI *n* = 161	[kg/m^2^]	22.33 (4.34)	18.31 (2.82)	0.01
		Z-score	0.97 (1.32)	−0.05 (0.69)	0.01

## Data Availability

Raw data used in the publication are available in Supplementary Materials.

## Supplementary Materials

The following supporting information can be downloaded at: https://www.mdpi.com/article/10.3390/jcm12237197/s1.

## References

[1] Wiegman A. (2018). Lipid Screening, Action, and Follow-up in Children and Adolescents. Curr. Cardiol. Rep..

[2] Mundal L., Igland J., Ose L., Holven K.B., Veierød M.B., Leren T.P., Retterstøl K. (2016). Cardiovascular Disease Mortality in Patients with Genetically Verified Familial Hypercholesterolemia in Norway during 1992–2013. Eur. J. Prev. Cardiol..

[3] Nordestgaard B.G., Benn M. (2017). Genetic Testing for Familial Hypercholesterolaemia Is Essential in Individuals with High LDL Cholesterol: Who Does It in the World?. Eur. Heart J..

[4] Goldberg A.C., Hopkins P.N., Toth P.P., Ballantyne C.M., Rader D.J., Robinson J.G., Daniels S.R., Gidding S.S., de Ferranti S.D., Ito M.K. (2011). Familial Hypercholesterolemia: Screening, Diagnosis and Management of Pediatric and Adult Patients Clinical Guidance from the National Lipid Association Expert Panel on Familial Hypercholesterolemia. J. Clin. Lipidol..

[5] Myśliwiec M., Walczak M., Małecka-Tendera E., Dobrzańska A., Cybulska B., Filipiak K.J., Mazur A., Jarosz-Chobot P., Szadkowska A., Rynkiewicz A. (2013). Management in Familial Hypercholesterolaemia in Children and Adolescents. Position of the Lipid Expert Forum. Kardiol. Pol..

[6] Narverud I., Retterstøl K., Iversen P.O., Halvorsen B., Ueland T., Ulven S.M., Ose L., Aukrust P., Veierød M.B., Holven K.B. (2014). Markers of Atherosclerotic Development in Children with Familial Hypercholesterolemia: A Literature Review. Atherosclerosis.

[7] Nordestgaard B.G., Chapman M.J., Humphries S.E., Ginsberg H.N., Masana L., Descamps O.S., Wiklund O., Hegele R.A., Raal F.J., Defesche J.C. (2013). Familial Hypercholesterolaemia Is Underdiagnosed and Undertreated in the General Population: Guidance for Clinicians to Prevent Coronary Heart Disease. Eur. Heart J..

[8] Cohen H., Stefanutti C., Giacomo S.D., Morozzi C., Widhalm K., Bjelakovic B.B., Berni A., Martino F., Bosco G. (2021). Current Approach to the Diagnosis and Treatment of Heterozygote and Homozygous FH Children and Adolescents. Curr. Atheroscler. Rep..

[9] Dombalis S., Nash A. (2020). The Effect of Statins in Children and Adolescents With Familial Hypercholesterolemia: A Systematic Review. J. Pediatr. Health Care.

[10] Vuorio A., Kuoppala J., Kovanen P.T., Humphries S.E., Tonstad S., Wiegman A., Drogari E., Ramaswami U. (2019). Statins for Children with Familial Hypercholesterolemia. Cochrane Database Syst. Rev..

[11] Martin A.C., Gidding S.S., Wiegman A., Watts G.F. (2017). Knowns and Unknowns in the Care of Pediatric Familial Hypercholesterolemia. J. Lipid Res..

[12] Ramaswami U., Cooper J., Humphries S.E., Group F.P.R.S. (2017). The UK Paediatric Familial Hypercholesterolaemia Register: Preliminary Data. Arch. Dis. Child..

[13] Bogsrud M.P., Langslet G., Wium C., Johansen D., Svilaas A., Holven K.B. (2018). Treatment Goal Attainment in Children with Familial Hypercholesterolemia: A Cohort Study of 302 Children in Norway. J. Clin. Lipidol..

[14] Kavey R.-E.W., Manlhiot C., Runeckles K., Collins T., Gidding S.S., Demczko M., Clauss S., Harahsheh A.S., Mietus-Syder M., Khoury M. (2020). Effectiveness and Safety of Statin Therapy in Children: A Real-World Clinical Practice Experience. CJC Open.

[15] Luirink I.K., Wiegman A., Kusters D.M., Hof M.H., Groothoff J.W., de Groot E., Kastelein J.J.P., Hutten B.A. (2019). 20-Year Follow-up of Statins in Children with Familial Hypercholesterolemia. N. Engl. J. Med..

[16] Wiegman A., Gidding S.S., Watts G.F., Chapman M.J., Ginsberg H.N., Cuchel M., Ose L., Averna M., Boileau C., Borén J. (2015). Familial Hypercholesterolaemia in Children and Adolescents: Gaining Decades of Life by Optimizing Detection and Treatment. Eur. Heart J..

[17] Kułaga Z., Różdżyńska-Świątkowska A., Grajda A., Gurzkowska B., Wojtyło M., Góźdź M., Świąder-Leśniak A., Litwin M. (2015). Percentile charts for growth and nutritional status assessment in Polish children and adolescents from birth to 18 year of age. Stand. Med. Pediatr..

[18] Ramaswami U., Futema M., Bogsrud M.P., Holven K.B., van Lennep J.R., Wiegman A., Descamps O.S., Vrablik M., Freiberger T., Dieplinger H. (2020). Comparison of the Characteristics at Diagnosis and Treatment of Children with Heterozygous Familial Hypercholesterolaemia (FH) from Eight European Countries. Atherosclerosis.

[19] Hennig M., Brandt-Varma A., Wołoszyn-Durkiewicz A., Bautembach-Minkowska J., Buraczewska M., Świętoń D., Mickiewicz A., Rynkiewicz A., Gruchała M., Limon J. (2020). Monitoring the Effects of Hypolipidemic Treatment in Children with Familial Hypercholesterolemia in Poland. Life.

[20] Lehtovirta M., Matthews L.A., Laitinen T.T., Nuotio J., Niinikoski H., Rovio S.P., Lagström H., Viikari J.S.A., Rönnemaa T., Jula A. (2021). Achievement of the Targets of the 20-Year Infancy-Onset Dietary Intervention—Association with Metabolic Profile from Childhood to Adulthood. Nutrients.

[21] Michos E.D., McEvoy J.W., Blumenthal R.S. (2019). Lipid Management for the Prevention of Atherosclerotic Cardiovascular Disease. N. Engl. J. Med..

[22] Cuchel M., McGowan M.P. (2021). Familial Hypercholesterolaemia: Too Many Lost Opportunities. Lancet.

[23] Langslet G., Johansen A.K., Bogsrud M.P., Narverud I., Risstad H., Retterstøl K., Holven K.B. (2021). Thirty Percent of Children and Young Adults with Familial Hypercholesterolemia Treated with Statins Have Adherence Issues. Am. J. Prev. Cardiol..

[24] Stroes E.S., Thompson P.D., Corsini A., Vladutiu G.D., Raal F.J., Ray K.K., Roden M., Stein E., Tokgözoğlu L., Nordestgaard B.G. (2015). Statin-Associated Muscle Symptoms: Impact on Statin Therapy—European Atherosclerosis Society Consensus Panel Statement on Assessment, Aetiology and Management. Eur. Heart J..

[25] Cai T., Abel L., Langford O., Monaghan G., Aronson J.K., Stevens R.J., Lay-Flurrie S., Koshiaris C., McManus R.J., Hobbs F.D.R. (2021). Associations between Statins and Adverse Events in Primary Prevention of Cardiovascular Disease: Systematic Review with Pairwise, Network, and Dose-Response Meta-Analyses. BMJ.

[26] Medeiros A.M., Alves A.C., Aguiar P., Bourbon M., on behalf of the Pediatric Investigators of the Portuguese Familial Hypercholesterolemia Study (2014). Cardiovascular Risk Assessment of Dyslipidemic Children: Analysis of Biomarkers to Identify Monogenic Dyslipidemia. J. Lipid Res..

[27] Vallejo-Vaz A.J., Stevens C.A.T., Lyons A.R.M., Dharmayat K.I., Freiberger T., Hovingh G.K., Mata P., Raal F.J., Santos R.D. (2021). Global Perspective of Familial Hypercholesterolaemia: A Cross-Sectional Study from the EAS Familial Hypercholesterolaemia Studies Collaboration (FHSC). Lancet.

[28] Diamond D.M., Alabdulgader A.A., de Lorgeril M., Harcombe Z., Kendrick M., Malhotra A., O’Neill B., Ravnskov U., Sultan S., Volek J.S. (2020). Dietary Recommendations for Familial Hypercholesterolaemia: An Evidence-Free Zone. BMJ Evid. Based Med..

[29] Groselj U., Wiegman A., Gidding S.S. (2022). Screening in Children for Familial Hypercholesterolaemia: Start Now. Eur. Heart J..

